# Case of Supposed Chronic Arachnitis

**Published:** 1830-07-01

**Authors:** 


### Case of supposed Chronic Arachnitis.

The following case is related by Dr. La Roche in our valuable trans-Atlantic contemporary, the North American Medical and Surgical Journal, for January of the present year.

Mr. E.G. æt. 60, of muscular frame and nervous temperament had long been subject to dyspeptic and hypochondriacal symptoms, and attacks resembling hysteria. His habits of life were, Dr. La Roche believed, regular, but six or seven years ago he was seized with an unusually severe paroxysm of nervous irritation, accompanied with slight delirium. A year ago when in Holland he had a still more violent attack, which would seem to have been considered as allied to delirium tremens, for it was treated successfully by opium. From this time till early in October Mr. G. enjoyed tolerable health, but he was then observed by his friends to become morose, avoid company, evince an occasional loss of memory, and complain of rheumatic pains in his lower extremities.

"At last, on the 19th of October, the explosion took place. I was called to see him at twelve o'clock of that day, and found him as follows: The muscles of his extremities were all in motion; he was unable, on this account, to remain on his feet or to walk. He could hold nothing in his hands, and it was with the utmost difficulty I could steady one of his arms sufficiently to feel his pulse. While holding my finger on the artery I could perceive a vibratory and continual motion in the muscles. His mind was perfectly sound, though his speech was rather more abrupt than usual. He had not slept the night before, and attributed all this agitation and a little heaviness over the eyes to this circumstance. His eyes were not affected by light: the head and indeed every part of the body was free from pain; but on sitting in bed he experienced some giddiness. The tongue was of a natural colour on the sides and tip, partaking a little of the trembling and agitation of the other muscles, and was slightly furred in the centre. He complained of thirst—loss of appetite; pulse about eighty, and rather hard and full; skin covered in parts with perspiration, but of natural temperature. He conversed gaily about his agitation, and related to me all I have stated above concerning his former attacks."

The patient objected to depletion and some opening medicine with abstinence were ordered, followed by mustard pediluvia, and night pills of camphor and acetate of morphia. On the next day he was so much better as to be found by the physician reading the newspaper, but the agitation of the muscles still remained. The sedatives were repeated, but towards evening he felt his hallucinations coming on, and accordingly next day they were fully established. Troubled images of dogs, cats, and men floated almost constantly before his eyes, the pulse became active and tense, and the tongue foul. Like persons affected with the mania à potu there was an odd mixture in the case of positive aberration and a knowledge of the unreal nature of his fantastic imagery. Depletion was refused, and infusion of senna with valerian was employed as a purge, with the application of cold to the head. The medicine operated well, and the patient appeared to be better, yet an oddness of manner remained, and thirty grains of assafœtida with two of acetate of morphia were prescribed, one half to be taken at bedtime and the other half in an hour if sleep was not procured. He retired to his room, but at 3, a.m. next morning jumped out of his window on the third floor, having previously locked his door for that purpose. Another physician prescribed a dose of laudanum after this occurrence, he fell into a quiet sleep, but this was even longer than the dreamer Nourjahad's, for he never woke again!

"On opening the cranium a large quantity of fluid escaped. The arachnoid on both parietal, as well as on the upper regions, was found thickened, and in some places slightly opaque. Other parts of this membrane, as also the piamater throughout, were highly vascular, and between them there was discovered a considerable serous effusion. The substance of the brain was much softer and more vascular than usual. The ventricles contained a large quantity of fluid, and were, especially the left, morbidly enlarged ; so much so indeed, that Dr. HORNER and the rest of us were inclined to believe the subject must have suffered at one time from a chronic hydrocephalus. The cerebellum was also very vascular, but presented no unusual appearance. At the lower edge of the falx the dura mater was found ossified in a circumscribed spot.

" *Back*—On making an incision over the spine, from the neck to the sacrum, a considerable effusion of blood was seen at the lower part of the spine and beginning of the sacrum, not perceptible by any discolouration or swelling externally on the skin. No fracture of the spine could be discovered, and the spinal cord was found healthy throughout.

" *Abdomen*. On raising the intestines, a very extensive effusion of blood was visible under the peritoneum. This cut, the blood was seen partially coagulated over the region of the iliac veins, extending somewhat upward ; but more particularly down into the cavity of the pelvis. No rupture of a large vessel was perceptible, so that we were led to infer that the blood proceeded from the rupture of several small veins.

" The stomach was somewhat distended, and contained a quantity of fluid, which, from its appearance and smell, could be easily recognized to be the wine and water and laudanum that had been administered an hour before death. The inner surface of this viscus was of a light ash colour, and the mucous coat so softened in its whole extent, that it was with the greatest ease scraped off with the handle of a scalpel. A portion of the mesentery was ruptured, and an effusion of blood was found between its laminæ. The liver was white and small.

" *Thorax*.—A large ecchymosis was found in the substance of the diaphragm. The right lung was healthy in its whole extent. In the anterior portion of the left, a cicatrix of the size of half a dollar was discovered ; around it a few aborted tubercles were situated. On neither side was there any adhesion of the pleura."

Dr. La Roche in his remarks on the preceding case, rather coarsely expresses his astonishment that it should " never have entered the heads of this patient's physicians for a minute," that the symptoms depended on an inflammation of the meninges. " Yet dissection," says the Doctor, " revealed to us the existence of a chronic inflammation of the arachnoid, which in all probability was the result of the acute forms of the disease supervening at every repetition of the complaint." Now we are by no means so certain of the truth of these statements as Dr. La Roche, for neither are the evidences of chronic inflammation of the arachnoid indisputable, nor the occasional attacks of acute inflammation of the same undeniable. An increased quantity of serous fluid, and that to a considerable amount, between the arachnoid and pia mater may exist without any inflammation of either, and for the confirmation of this statement we appeal to all who are in the habit of opening many bodies. We affirm that in two cases out of four, an effusion of serum to some extent between the membranes in question will be found after death. Neither is the enlargement of the cavities of the ventricles, a positive sign of arachnitis, on the contrary such enlargement is most frequent in dropsical patients, and independent in many instances of any thing a-kin to inflammatory action.* We demur then, for these anatomical reasons, to the opinion that arachnitis *necessarily* existed, and our doubts are increased by the history and progress of the case. We grant that there was an excitement kindled up from time to time within the head, but we question

* The violent death of the patient would prevent our pinning any faith on the partial vascularities observed in the cranium.

whether the symptoms would not have been exasperated by *active* depletion. The tendency to *serous* collection within the ventricles and between the membranes is certainly given by maniacal affections, by delirium tremens, and maladies of that class, and experience has proved that though local depletion and counter-irritation is often beneficial, the bold treatment adapted for phrenitis is positively mischievous. One word with regard to purgation. We have witnessed violent attacks of maniacal delirium threatening to end fatally arrested by a sharp and drastic purge of calomel and scammony. Of course there is no novelty in this observation, as it was the purgative quality of hellebore, that established its fame for the cure of madness in the ancient world.

